# Coronary Artery Plaque Characteristics Associated With Adverse Outcomes in the SCOT-HEART Study

**DOI:** 10.1016/j.jacc.2018.10.066

**Published:** 2019-01-29

**Authors:** Michelle C. Williams, Alastair J. Moss, Marc Dweck, Philip D. Adamson, Shirjel Alam, Amanda Hunter, Anoop S.V. Shah, Tania Pawade, Jonathan R. Weir-McCall, Giles Roditi, Edwin J.R. van Beek, David E. Newby, Edward D. Nicol

**Affiliations:** a University of Edinburgh/British Heart Foundation Centre for Cardiovascular Science, Edinburgh, United Kingdom; b Edinburgh Imaging Facility QMRI, University of Edinburgh, Edinburgh, United Kingdom; c Christchurch Heart Institute, University of Otago, Christchurch, New Zealand; d University of Cambridge School of Clinical Medicine, Biomedical Research Centre, University of Cambridge, Cambridge, United Kingdom; e Institute of Clinical Sciences, University of Glasgow, Glasgow, United Kingdom; f Royal Brompton and Harefield NHS Foundation Trust Departments of Cardiology and Radiology, London, United Kingdom; g National Heart and Lung Institute, Faculty of Medicine, Imperial College, London, United Kingdom

**Keywords:** atherosclerotic plaque, computed tomography, coronary angiography, coronary artery disease, AU, Agatston units, CI, confidence interval, CT, computed tomography, CTA, computed tomography angiography, HR, hazard ratio, IQR, interquartile range

## Abstract

**Background:**

Unlike most noninvasive imaging modalities, coronary computed tomography angiography can characterize subtypes of atherosclerotic plaque.

**Objectives:**

The purpose of this study was to investigate the prognostic implications of adverse coronary plaque characteristics in patients with suspected coronary artery disease.

**Methods:**

In this SCOT-HEART (Scottish COmputed Tomography of the HEART Trial) post hoc analysis, the presence of adverse plaque (positive remodeling or low attenuation plaque), obstructive disease, and coronary artery calcification within 15 coronary segments was assessed on coronary computed tomography angiography of 1,769 patients who were followed-up for 5 years.

**Results:**

Among study participants (mean age 58 ± 10 years; 56% male), 608 (34%) patients had 1 or more adverse plaque features. Coronary heart disease death or nonfatal myocardial infarction was 3 times more frequent in patients with adverse plaque (n = 25 of 608 [4.1%] vs. n = 16 of 1,161 [1.4%]; p < 0.001; hazard ratio [HR]: 3.01; 95% confidence interval (CI): 1.61 to 5.63; p = 0.001) and was twice as frequent in those with obstructive disease (n = 22 of 452 [4.9%] vs. n = 16 of 671 [2.4%]; p = 0.024; HR: 1.99; 95% CI: 1.05 to 3.79; p = 0.036). Patients with both obstructive disease and adverse plaque had the highest event rate, with a 10-fold increase in coronary heart disease death or nonfatal myocardial infarction compared with patients with normal coronary arteries (HR: 11.50; 95% CI: 3.39 to 39.04; p < 0.001). However, these associations were not independent of coronary artery calcium score, a surrogate measure of coronary plaque burden.

**Conclusions:**

Adverse coronary plaque characteristics and overall calcified plaque burden confer an increased risk of coronary heart disease death or nonfatal myocardial infarction. (Scottish COmputed Tomography of the HEART Trial [SCOT-HEART]; NCT01149590)

The investigation of patients with suspected coronary artery disease has previously focused on functional assessments that attempt to identify the presence of myocardial ischemia as a downstream surrogate marker of proximal coronary artery stenoses. In contrast, noninvasive imaging with coronary computed tomography angiography (CTA) has the ability to provide precise structural information of the coronary artery wall, and can assess for the presence and constituents of atherosclerotic plaque even in the absence of flow-limiting disease.

Pathological studies in patients with myocardial infarction have identified an association between plaque rupture and adverse plaque characteristics that includes positive remodeling, a large necrotic core, microcalcification, and a thin fibrous cap (1). Correlates of these features have been described for noninvasive imaging with coronary CTA and include the presence of positive remodeling, low attenuation plaque, spotty calcification, and the “napkin ring” sign (2). These plaque characteristics are associated with an increased risk of subsequent acute coronary syndromes 2, 3, 4. Recent data have suggested that positive remodeling and low attenuation plaque in particular provide the most useful prognostic information 2, 5, although it remains unclear whether this is of incremental value to traditional cardiovascular risk factors or coronary plaque burden.

In the SCOT-HEART (Scottish COmputed Tomography of the HEART) prospective, multicenter, randomized controlled trial of patients with stable chest pain, the addition of coronary CTA to routine care led to improved diagnostic certainty and patient care that ultimately reduced the rate of coronary heart disease death or nonfatal myocardial infarction 6, 7, 8. These benefits were largely attributable to subsequent changes in patient management and treatment, which had been guided by the presence of obstructive or nonobstructive coronary artery disease as determined by coronary CTA. However, it may be that further risk stratification and targeted intensification of therapy in patients with adverse plaque characteristics could achieve additional benefits that go beyond the presence of obstructive or nonobstructive coronary artery disease.

In this secondary analysis of the SCOT-HEART trial, we aimed to determine the extent of adverse coronary artery plaque characteristics on coronary CTA and their association with subsequent clinical outcomes. If confirmed, the identification of these coronary artery plaque characteristics may help risk stratification and guide the intensity of therapy.

## Methods

### Study design

The SCOT-HEART trial was a multicenter randomized controlled trial of coronary CTA in outpatients with suspected angina pectoris due to coronary artery disease (9). The primary results have been reported previously 6, 7, 8. This paper presents a secondary post hoc analysis of the SCOT-HEART study.

### Participants

In brief, 4,146 patients who attended the cardiology outpatient clinic were randomized to standard care alone or standard care plus coronary CTA, and were followed up for symptoms, management, and outcomes. Of the 2,073 participants who were randomized to the intervention arm, 1,778 underwent coronary CTA. Coronary CTA and noncontrast imaging for calcium scoring was performed as described previously (7). Cardiovascular risk was assessed using the ASSIGN (ASsessing cardiovascular risk using Scottish Intercollegiate Guideline Network guidelines) score. This score has been validated for the Scottish population and, in addition to traditional cardiovascular risk factors, incorporates social deprivation and family history of cardiovascular disease (10).

### Assessment of computed tomography images

Coronary artery calcium score was assessed on noncontrast computed tomography (CT) using the Agatston scoring method as previously described (11). Coronary artery calcium scores were classified into 4 categories (0, 1 to 99, 100 to 399, and ≥400 Agatston units [AU]).

On coronary CTA images, per-segment assessment of atherosclerotic plaque was performed using a 15-segment model by 6 trained observers, with complex cases classified by consensus (12). Normal coronary arteries were defined by the absence of obstructive or nonobstructive atherosclerotic plaque. Nonobstructive coronary artery disease was defined by the presence of atherosclerotic plaque occupying a cross-sectional area stenosis ≤70%. Obstructive coronary artery disease was defined as a stenosis >70% in 1 or more major epicardial vessel or >50% in the left main stem. Observer agreement for the identification of coronary artery disease on coronary CTA and coronary artery calcium score in the SCOT-HEART study has previously been shown to be excellent (13).

**Figure 1:**
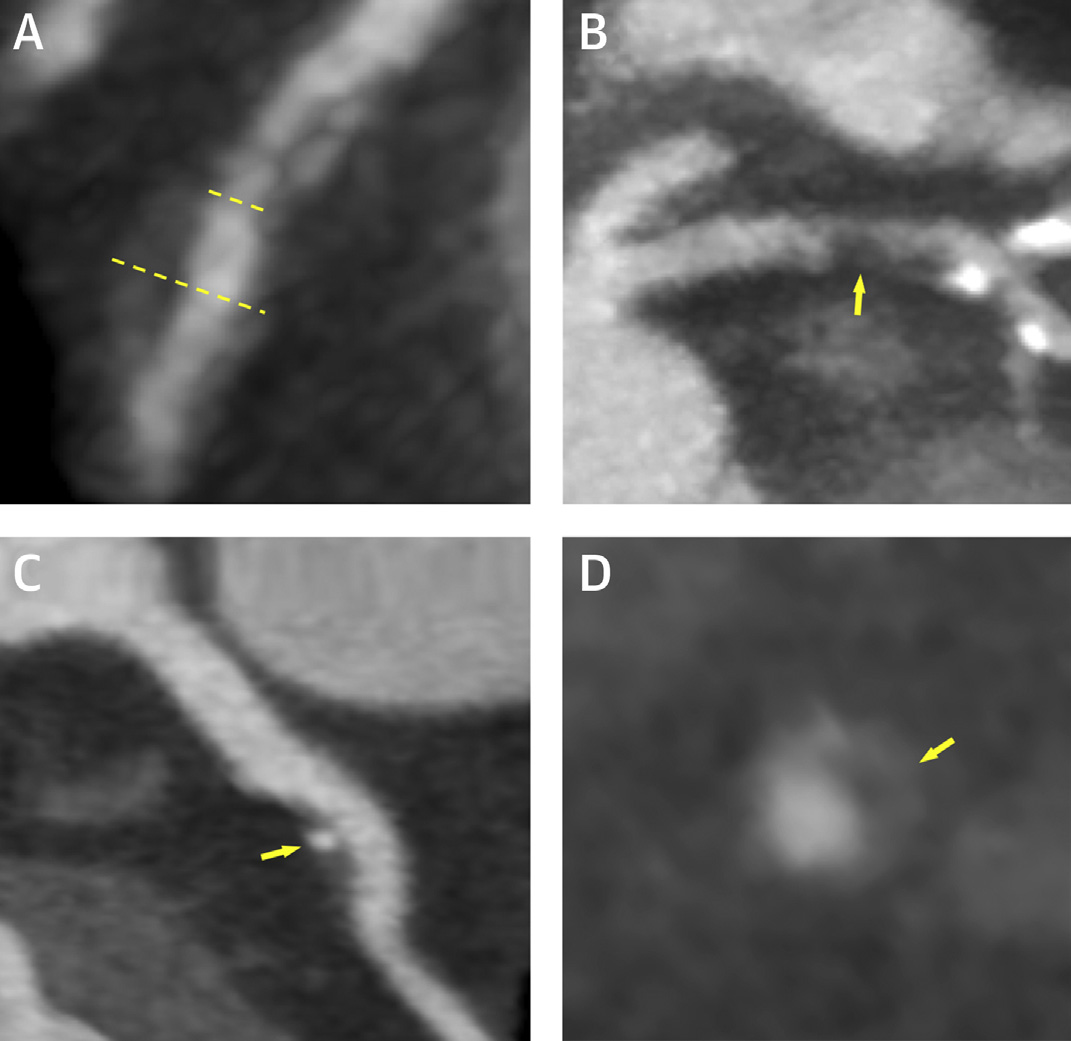
Coronary Plaque Characteristics Identified on Computed Tomography Coronary Angiography Coronary atherosclerotic plaque features detected using computed tomography coronary angiography including **(A)** positive remodeling, **(B)** low-attenuation plaque, **(C)** spotty calcification, and **(D)** the “napkin ring” sign. Positive remodeling **(A)** was defined as an outer vessel diameter **(long dashed line)** that was 10% greater than the mean diameter of the segments immediately proximal **(short dashed line)** and distal to the plaque. Low-attenuation plaque **(B)** was defined as a focal central area of plaque with an attenuation density of <30 Hounsfield Units **(yellow arrow)**. Spotty calcification **(C)** was defined as focal calcification within the coronary artery wall that measured <3 mm in maximum diameter **(yellow arrow)**. The “napkin ring” sign **(D)** was defined as a central area of low-attenuation plaque with a peripheral rim of high attenuation **(yellow arrow)**.

For each segment, the presence or absence of 4 coronary artery plaque characteristics was assessed (Figure 1): positive remodeling, low attenuation plaque, spotty calcification, and the “napkin ring” sign. Positive remodeling was defined as an outer vessel diameter that was 10% greater than the mean of the diameter of the segments immediately proximal and distal to the plaque (3). Low-attenuation plaque was defined as a focal central area of plaque with an attenuation density of <30 Hounsfield Units (14). Spotty calcification was defined as focal calcification within the coronary artery wall <3 mm in maximum diameter (3). The “napkin ring” sign was defined, as previously described, as a central area of low-attenuation plaque that had a peripheral rim of high attenuation (15). Observer agreement for the assessment of coronary artery plaque characteristics has overall been shown to be fair (16).

An individual adverse plaque was defined as one with positive remodeling or low-attenuation plaque, as described previously by Motoyama et al. (3). Patients with 1 or more adverse plaques were defined as having adverse plaque.

### Clinical management and outcomes

Outcome information including invasive coronary angiography and coronary revascularization (percutaneous coronary intervention or coronary artery bypass graft surgery) was obtained from the electronic Data Research and Innovation Service of the National Health Service (NHS) Scotland and, where appropriate, confirmed by review of the patient health records. Categorization of outcomes was performed blinded to the coronary CTA or other study data. The primary clinical endpoint for this substudy was the occurrence of coronary heart disease death or nonfatal myocardial infarction. Outcome data were updated in March 2018 (6).

### Statistical analysis

Statistical analysis was performed using R version 3.5.0 (R Foundation for Statistical Computing, Vienna, Austria). Normally distributed quantitative variables are presented with mean ± SD. Non-normally distributed data are presented with median and interquartile range (IQR). Statistical significance was assessed using Pearson’s chi-square test, Fisher exact test, Student’s *t*-test, Mann-Whitney *U* test, Kruskal-Wallis test, or analysis of variance as appropriate. Outcome data investigating whether the presence of adverse plaque characteristics predicted coronary heart disease death or nonfatal myocardial infarction in patients with nonobstructive disease were analyzed using Cox proportional hazards regression and presented graphically using cumulative incidence plots. A time-to-event analysis was performed using coronary heart disease death or nonfatal myocardial infarction as the endpoint, with the primary independent variable being the presence or absence of adverse plaque on CT. Deaths not classified as coronary heart disease deaths were censored for both the Cox regression analysis and the cumulative incidence plots. Hazard ratios (HRs) and 95% confidence intervals (CIs) are presented. Coronary artery calcium score and cardiovascular risk score were log transformed for analysis (log base 2 of 1 plus the parameter). The variables included in the univariable and multivariable analysis are shown in the relevant tables. Variables were included in multivariable analysis if they were statistically significant on univariable analysis. The assumption of proportional hazards was checked using SPSS. A statistically significant difference was defined as a 2-sided p value <0.05.

## Results

Of the 1,778 individuals who underwent a coronary CTA, 1,769 participants had images that were available and of suitable quality for analysis (Online Figure 1). Patients had a mean age of 58 ± 10 years, and 56% were male with a range of cardiovascular risk factors and symptoms (Table 1). Of these patients, 37% (n = 646) had normal coronary arteries, 38% (n = 671) had nonobstructive coronary artery disease, and 26% (n = 452) had obstructive coronary artery disease. Coronary heart disease death or nonfatal myocardial infarction occurred in 41 patients (2.3%) over a median of 4.7 years of follow-up (IQR: 4.0 to 5.7 years).

**Table 1:** Characteristics of Study Participants

	All Participants (N = 1,769)	Participants Without Adverse Plaque (n = 1,161)	Participants With Adverse Plaque (n = 608)	p Value
Male	997 (56)	545 (47)	452 (74)	**<0.001**
Age, yrs	57.6 ± 9.5	55.9 ± 9.8	60.8 ± 7.8	**<0.001**
Body mass index, kg/m^2^	29.6 ± 5.5	30.0 ± 5.8	28.8 ± 4.8	**<0.001**
Atrial fibrillation	34 (1.9)	20 (1.7)	14 (2.3)	0.399
Smoking status				**<0.001**
Current smoker	330 (19)	208 (18)	122 (20)	
Ex-smoker	593 (34)	357 (31)	236 (39)	
Nonsmoker	845 (48)	595 (51)	250 (41)	
Hypertension	608 (35)	373 (32)	235 (39)	**0.005**
Diabetes	196 (11)	128 (11)	68 (11)	0.919
Family history of CHD	765 (43)	507 (44)	258 (42)	0.552
Previous CHD	178 (10)	75 (6.5)	103 (17)	**<0.001**
Previous PAD	31 (1.8)	19 (1.6)	12 (2)	0.608
Anginal symptoms				**<0.001**
Typical angina	654 (37)	347 (30)	307 (51)	
Atypical angina	432 (24)	293 (25)	139 (23)	
Nonanginal	683 (39)	521 (45)	162 (27)	
ASSIGN	17.9 ± 11.0	16.1 ± 10.9	21.4 ± 10.6	**<0.001**
Coronary artery calcium score (Agatston units)	21 [0–230]	0 [0–34]	281 [89–775]	**<0.001**

Values are n (%), mean ± SD, or median [interquartile range]. Values in **bold** indicate statistical significance.

ASSIGN = ASsessing cardiovascular risk using Scottish Intercollegiate Guidelines Network guidelines; CHD = coronary heart disease; PAD = peripheral arterial disease.

### Plaque characteristics

Coronary CTA characteristics of atherosclerotic plaque were assessed in 26,535 segments in 1,769 patients. This demonstrated positive remodeling in 1,163 segments (4.4%), low attenuation plaque in 213 segments (0.8%), spotty calcification in 472 segments (1.8%), and the napkin ring sign in 78 segments (0.3%). All plaque characteristics were more common in the proximal coronary artery segments, in particular, the proximal left anterior descending coronary artery (Table 2, Online Tables 1 to 4).

**Table 2:** Frequency of Plaque Characteristics in Each of the 15 Coronary Artery Segments

	Adverse Plaque∗	Positive Remodeling	Low-Attenuation Plaque	Spotty Calcification	Napkin Ring Sign
Left main stem	60 (3.4)	59 (3.3)	12 (0.7)	13 (0.7)	5 (0.3)
Prox LAD	388 (21.9)	385 (21.8)	78 (4.4)	117 (6.6)	33 (1.9)
Mid LAD	234 (13.2)	224 (12.7)	50 (2.8)	84 (4.7)	13 (0.7)
Dist LAD	21 (1.2)	19 (1.1)	5 (0.3)	14 (0.8)	1 (0.1)
D1	19 (1.1)	19 (1.1)	1 (0.1)	20 (1.1)	1 (0.1)
D2	5 (0.3)	5 (0.3)	0 (0.0)	8 (0.5)	0 (0.0)
Prox Cx	109 (6.2)	106 (6.0)	18 (1.0)	39 (2.2)	6 (0.3)
OM1	27 (1.5)	26 (1.5)	4 (0.2)	13 (0.7)	1 (0.1)
AV Cx	11 (0.6)	11 (0.6)	1 (0.1)	4 (0.2)	1 (0.1)
OM 2	1 (0.1)	1 (0.1)	0 (0.0)	2 (0.1)	0 (0.0)
Mid dist Cx	5 (0.3)	5 (0.3)	0 (0.0)	1 (0.1)	0 (0.0)
Prox RCA	116 (6.6)	112 (6.3)	20 (1.1)	56 (3.2)	6 (0.3)
Mid RCA	132 (7.5)	130 (7.3)	19 (1.1)	63 (3.6)	10 (0.6)
Distal RCA	51 (2.9)	51 (2.9)	4 (0.2)	25 (1.4)	1 (0.1)
RCA/CX PD	10 (0.6)	10 (0.6)	1 (0.1)	13 (0.7)	0 (0.0)
Per-vessel analysis†					
LAD	527 (29.8)	517 (29.2)	129 (7.3)	203 (11.5)	52 (2.9)
LCX	139 (7.9)	137 (7.7)	22 (1.2)	52 (2.9)	8 (0.5)
RCA	222 (12.5)	219 (12.4)	41 (2.3)	112 (6.3)	16 (0.9)

Values are n (%).

AV = atrioventricular; Cx = circumflex; D1 = first diagonal; D2 = second diagonal; LAD = left anterior descending; LM = left main; OM1 = obtuse marginal; PD = posterior descending; Prox = proximal; RCA = right coronary artery.

∗ Positive remodeling or low-attenuation plaque.

† 1 or more plaque features per-vessel.

**Figure 2:**
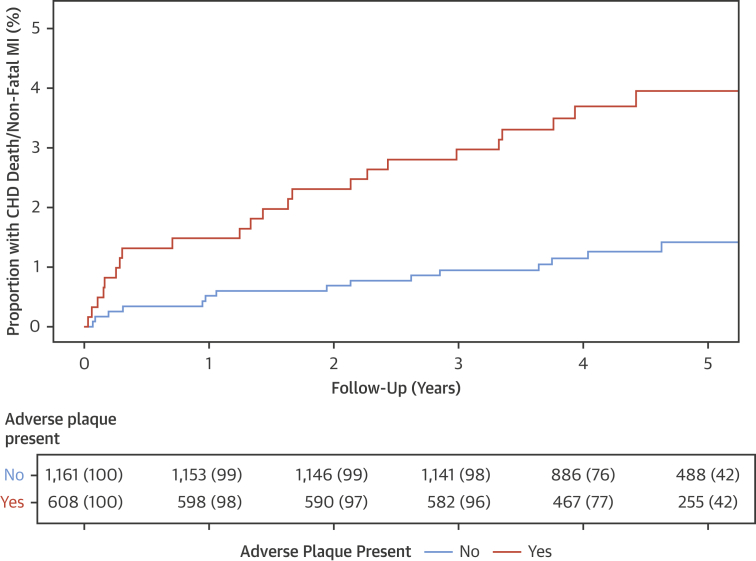
Coronary Heart Disease Death or Nonfatal Myocardial Infarction Across the Total Cohort in Patients With and Without Adverse Plaque The effect of the presence of 1 or more adverse plaques (defined by the presence of low attenuation or positive remodeling) on subsequent coronary heart disease (CHD) death or nonfatal myocardial infarction (MI). Cumulative incidence plot for patients with and without adverse plaque features.

Adverse plaques were present in 608 (34%) patients. Patients with adverse plaques were older, had a higher body mass index, and were more likely to be male. They were also more likely to have a previous history of coronary heart disease or hypertension, to be a smoker, or to have typical angina (Table 1, Online Table 5). Patients with adverse plaques had a higher cardiovascular risk score and higher calcium score. Coronary heart disease death or nonfatal myocardial infarction was 3 times more frequent in patients with adverse plaque compared to those without (n = 25 of 608 [4.1%] vs. n = 16 of 1,161 [1.4%]; p < 0.001; HR: 3.01; 95% CI: 1.61 to 5.63; p = 0.001) (Figure 2). Most events occurred in patients with adverse plaques in the proximal segments rather than the mid and distal segments (n = 23 of 489 [4.7%] vs. n = 2 of 119 [1.7%]; p = 0.197). Adverse plaque features appeared to have a greater prognostic value in women and those under the age of 60 years, with a body mass index >30 kg/m^2^ or with a lower cardiovascular risk score, but these differences were not statistically significant (Online Table 6). The presence of 1 or more segments of spotty calcification or the napkin ring sign was not associated with a difference in coronary heart disease death or nonfatal myocardial infarction on per-patient assessment (Online Tables 3 and 7).

### Coronary artery stenosis

Patients with obstructive coronary artery disease were twice as likely to experience coronary heart disease death or nonfatal myocardial infarction (n = 22 of 452 [4.9%]) than patients with nonobstructive disease (n = 16 of 671 [2.4%]; chi-square p = 0.024; HR: 1.99; 95% CI: 1.05 to 3.79; p = 0.036). Patients with obstructive coronary artery disease were also more likely to undergo coronary revascularization (n = 215 of 452 [48%] vs. n = 35 of 671 [5%]; p < 0.001) and be prescribed preventative medications at 6 weeks (n = 427 of 452 [95%] vs. n = 538 of 671 [80%]; p < 0.001) than patients with nonobstructive disease.

**Figure 3:**
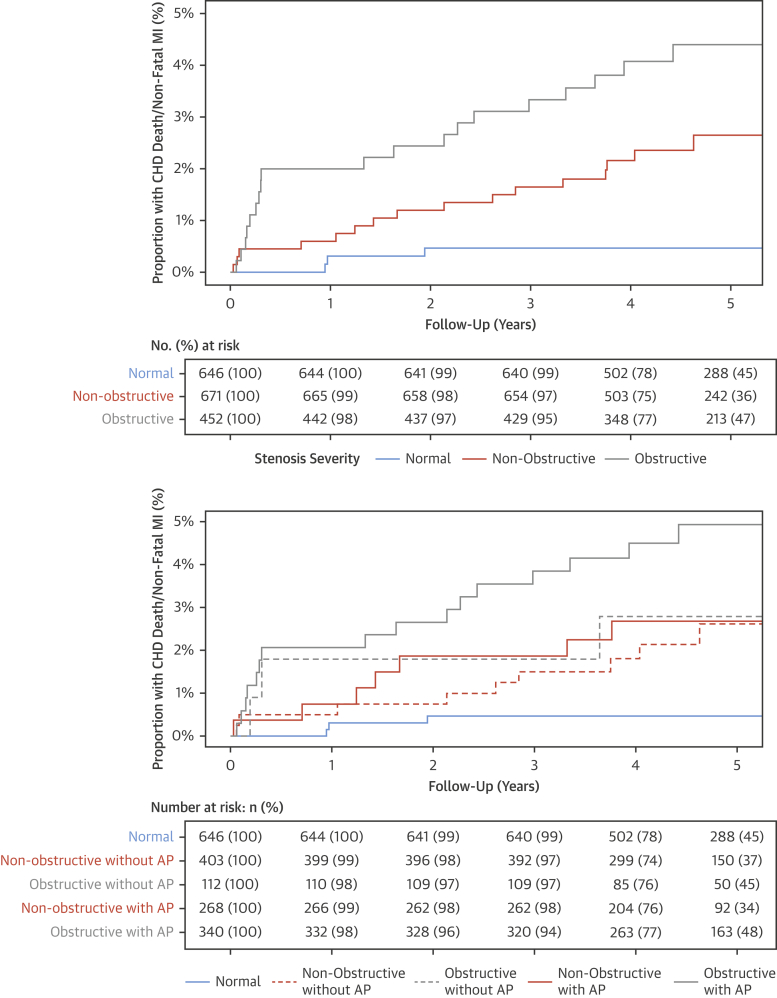
Coronary Heart Disease Death and Nonfatal Myocardial Infarction in Patients With Different Stenosis Severity and Coronary Artery Plaque Characteristics Cumulative incidence plot for coronary heart disease death and nonfatal myocardial infarction in patients **(top)** with normal coronary arteries, nonobstructive disease, and obstructive disease and in **(bottom)** normal coronary arteries, nonobstructive disease with and without adverse plaque characteristics, and obstructive disease with and without adverse plaque features. AP = adverse plaque; other abbreviations as in Figure 2.

Adverse plaques were observed in 40% of patients with nonobstructive disease (n = 268 of 671) and three-quarters of patients with obstructive coronary artery disease (n = 340 of 452). Patients with both obstructive disease and adverse plaque had the worst outcome during follow-up (Figure 3). Although the presence of adverse plaque is suggestive of a worse prognosis at 2 years in patients with nonobstructive disease, at 5 years the outcomes of patients with nonobstructive disease and obstructive disease without adverse plaque were similar (Figure 3). Indeed, compared with patients with normal coronary arteries, patients with obstructive disease and adverse plaque had a >10-fold increase in the rate of coronary heart disease death or nonfatal myocardial infarction at 5 years (Table 3).

**Table 3:** Univariable and Multivariable Analysis for Coronary Heart Disease Death or Nonfatal Myocardial Infarction Compared With Patients With Normal Coronary Arteries

	Univariable Analysis	p Value	Multivariate Analysis∗	p Value
Nonobstructive disease without adverse plaque†	4.95 (1.34–18.29)	**0.016**	2.79 (0.64–12.18)	0.172
Nonobstructive disease with adverse plaque†	5.81 (1.50–22.46)	**0.011**	2.67 (0.53–13.43)	0.234
Obstructive disease without adverse plaque†	7.73 (1.73–34.54)	**0.007**	3.20 (0.52–19.61)	0.208
Obstructive disease with adverse plaque†	11.50 (3.39–39.04)	**<0.001**	4.10 (0.76–22.0)	0.100
Coronary artery calcium score‡	1.23 (1.13–1.35)	**<0.001**	1.12 (0.98–1.28)	0.107
Cardiovascular risk score§	1.65 (1.13–2.41)	**0.01**	1.10 (0.71–1.70)	0.673

Values are hazard ratio (95% confidence interval). Values in **bold** indicate statistical significance.

∗ Log rank statistic 27.29, p < 0.001; Harrell’s C-statistic 0.728 (standard error = 0.046).

† Adverse plaque defined as the presence of positive remodeling or low-attenuation plaque.

‡ Per doubling of coronary artery calcium score.

§ ASSIGN score, per doubling of cardiovascular risk score.

### Coronary artery calcium score

**Figure 4:**
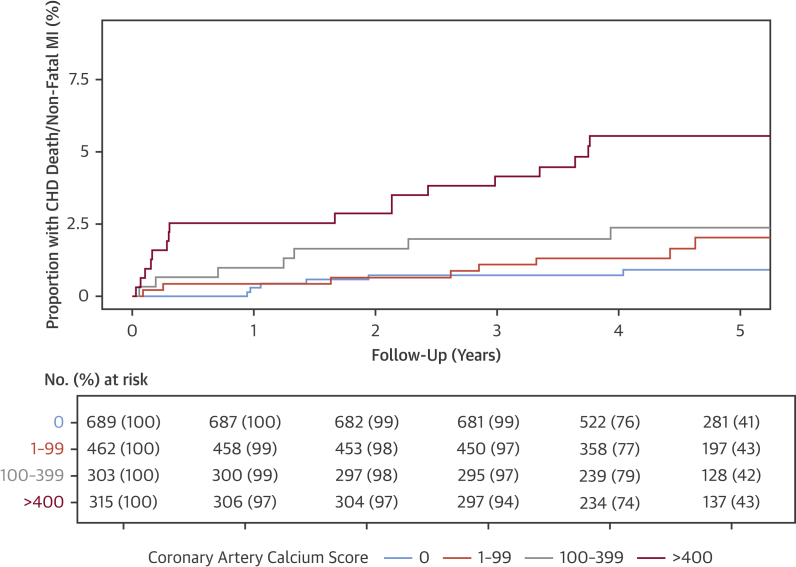
Coronary Heart Disease Death or Nonfatal Myocardial Infarction in Patients With Different Severity of Coronary Artery Calcification Cumulative incidence plots of the effect of different severity of coronary artery calcification on subsequent coronary heart disease death or nonfatal myocardial infarction. Abbreviations as in Figure 2.

Patients with a coronary artery calcium score ≥1,000 AU had a 13-fold increase in coronary heart disease death or nonfatal myocardial infarction compared with patients without coronary artery calcification. There was a clear gradation of risk, with calcification associated with increasing risk (Figure 4). Patients with obstructive coronary artery disease had an 8-fold higher coronary artery calcium score than patients with nonobstructive coronary artery disease (435 AU [IQR: 138 to 1,127 AU] vs. 54 AU [IQR: 12 to 190 AU]; p < 0.001). In multivariable analysis, the presence of both adverse plaque and obstructive plaque were dependent on coronary artery calcium score as a predictor of coronary heart disease death or nonfatal myocardial infarction (Table 4, Online Table 8).

**Table 4:** Univariable and Multivariable Analysis for Coronary Heart Disease Death or Nonfatal Myocardial Infarction Across the Total Population

	Univariable Analysis	p Value	Multivariable Analysis∗	p Value
Adverse plaque†	3.01 (1.61–5.63)	**0.001**	1.18 (0.55–2.52)	0.671
Coronary artery calcium score‡	1.23 (1.13–1.35)	**<0.001**	1.17 (1.04–1.33)	**0.011**
Obstructive coronary artery disease	3.35 (1.81–6.19)	**<0.001**	1.36 (0.63–2.93)	0.439
Cardiovascular risk score§	1.65 (1.13–2.41)	**0.01**	1.14 (0.74–1.75)	0.563

Values are hazard ratio (95% confidence interval). Values in **bold** indicate statistical significance.

∗ Log rank statistic 26.74 (p < 0.001), Harrell’s C-statistic 0.723 (standard error = 0.046).

† Compared with patients without adverse plaque in the total population. Adverse plaque defined as the presence of positive remodeling or low-attenuation plaque.

‡ Per doubling of coronary artery calcium score.

§ ASSIGN score, per doubling of cardiovascular risk score.

There was no difference in the risk of events in patients with or without adverse plaque who had a CACS >100 AU. For patients with a CACS <100 AU, those with adverse plaque were at an increased risk of coronary heart disease death or nonfatal myocardial infarction compared with patients without adverse plaque (HR: 3.38; 95% CI: 1.13 to 10.08; p = 0.03).

## Discussion

In a large, multicenter, prospective study of coronary CTA, we demonstrated that adverse coronary plaque characteristics are associated with a tripling of the risk of coronary heart disease death or nonfatal myocardial infarction. The presence of obstructive disease was also a major predictor of risk, with the combination of adverse plaque with obstructive disease appearing to confer the greatest risk (Central Illustration). At 5 years, the risk of coronary heart disease death or nonfatal myocardial infarction was similar in patients with obstructive disease without adverse plaque and nonobstructive disease with or without adverse plaque. However, overall, the only independent predictor of risk was the coronary artery calcium score, a surrogate measure of overall plaque burden. These results suggest that although plaque composition and its hemodynamic consequences are associated with future myocardial infarction, the predominant factor governing patient outcomes is the burden of coronary atherosclerosis.

**Central Illustration:**
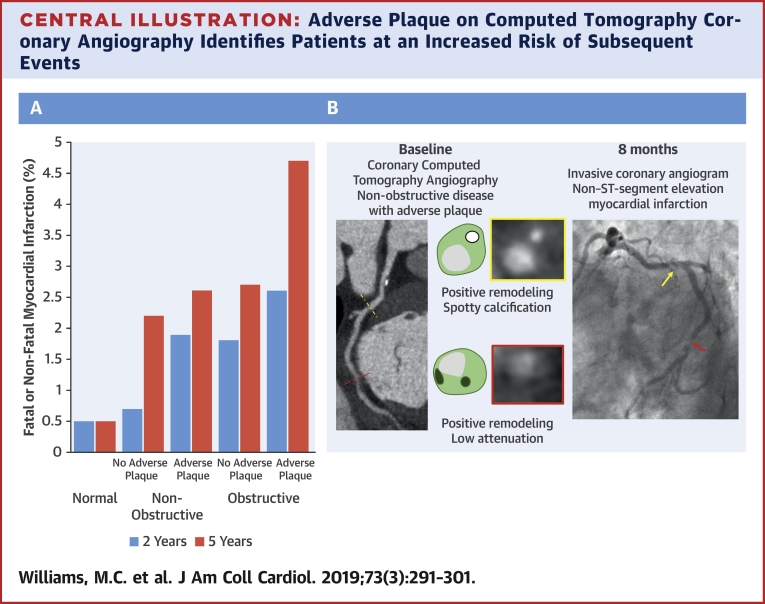
Adverse Plaque on Computed Tomography Coronary Angiography Identifies Patients at an Increased Risk of Subsequent Events **(A)** Bar graph of the frequency of coronary heart disease death or nonfatal myocardial infarction at 2 and 5 years for patients with normal coronary arteries and nonobstructive or obstructive disease with and without adverse plaque. **(B)** Coronary computed tomography angiography and invasive coronary angiography images from a patient with nonobstructive coronary artery disease who had a subsequent non–ST-segment elevation myocardial infarction. The **red/yellow dotted lines** and **arrows** correspond to the location of the plaques in the **red/yellow boxes**.

Adverse coronary plaque is associated with subsequent cardiovascular events in patients with stable coronary artery disease 2, 4, 5, 17. In keeping with previous work 2, 3, 17, 18, 19, we found that the plaque characteristics that were the most helpful in predicting future coronary events were positive remodeling and low-attenuation plaque. These CT characteristics are markers of pathological vulnerability (20), and are potential culprit lesions for subsequent acute coronary events. Motoyama et al. (2) identified that the presence of adverse plaque was predictive of acute coronary syndrome at 4 years in a study of 3,158 patients with 88 events (2.8%). In the PROMISE (Prospective Multicentre Imaging Study for Evaluation of Chest Pain) trial, adverse plaques were present in 15% of patients and were associated with an increased risk of the combined endpoint of death, myocardial infarction, or unstable angina (21). However, overall adverse plaques have a low positive predictive value for the identification of subsequent coronary events. This is in keeping with the known theory of continuous plaque remodeling, where adverse plaques may stabilize with or without subclinical rupture, rather than cause a clinically apparent acute coronary syndrome.

Acute myocardial infarction occurs when there is rupture or erosion of a coronary atherosclerotic plaque and there is associated thrombus formation causing vessel occlusion (22). The presence of pre-existing flow limitation due to obstructive atheroma is not a prerequisite for this process. Indeed, it should be remembered that the majority (∼80%) of myocardial infarctions are attributable to nonobstructive coronary plaques on antecedent angiography (23). Moreover, the risk of future myocardial infarction is similar whether the coronary plaque causes 50% to 85% or >85% luminal stenosis (24). A nested case-control study of patients without known coronary artery disease within the CONFIRM (Coronary CT Angiography Evaluation for Clinical Outcomes: An International Multicentre) registry identified that >65% of patients who developed acute coronary syndromes had nonobstructive disease on their baseline coronary CTA (25). This reflects the important nature of the biology of the atherosclerotic plaque rather than the functional consequence of luminal stenosis (26). For the prediction of subsequent coronary events, the presence of atherosclerotic plaque is more important than the presence of coronary stenoses. Indeed, in our study, patients with a combination of both obstructive coronary artery disease and adverse plaque had the worst outcomes. Moreover, patients with nonobstructive disease and adverse plaque had similar outcomes to patients with obstructive disease without adverse plaque. Thus, our study provides evidence that coronary artery disease should no longer be defined based on luminal severity, but instead by the volume and type of disease.

When examined across the cohort as a whole, adverse plaques did not provide independent prognostic information when CT calcium scoring was included. CT calcium scoring is a surrogate marker of plaque burden, and its prognostic utility has been established in large studies of symptomatic and asymptomatic patients 27, 28. It is likely that patients with higher plaque burden are more likely to have adverse plaques and are more likely to have severe coronary artery stenoses. Few studies have assessed the clinical implications of this overlap. A small study of 339 patients with suspected coronary artery disease found that both adverse plaques and CT calcium score were independent predictors of cardiac events at 2 years (29). Clearly, the greater the burden of disease, the greater the risk of events. However, for the individual patient, the assessment of coronary artery calcium alone is not sufficient to guide management, as the presence and volume of coronary artery calcification is not directly related to the degree of coronary artery stenosis and cannot inform on the pathophysiological status of the atherosclerotic plaque (30). Indeed, in the PROMISE study, coronary CTA provided better prognostic discrimination compared to coronary artery calcium score (31). Intriguingly, we also observed that adverse plaque was particularly discriminatory in patients with a low calcium score, reaffirming the concept that calcification is a healing or protective response to coronary atherosclerosis and that large areas of calcification are associated with plaque stabilization.

It is perhaps not surprising that the identification of plaque characteristics at a single time point by coronary CTA may not be sufficient to predict future events in long-term follow-up. Atherosclerotic plaque undergoes continuous remodeling driven by a variety of genetic and environmental factors, and we know that medications, such as statins, alter the constituents of atherosclerotic plaque (32). Autopsy studies show that atherosclerosis is present in young people (33) and nomograms for CT calcium score (28) and plaque burden (34) are in clinical use. Concerns have been raised about overmedicating patients with the routine use of preventative therapies for patients with nonobstructive disease (35). However, the event rate in patients with nonobstructive disease in our study highlights that atherosclerosis is not “normal.” Similarly, in the PROMISE study, coronary CTA identified patients with nonobstructive disease who were at risk of subsequent events (36). Future studies should assess whether the identification of coronary artery disease per se can guide treatment changes and improve outcomes for the wider population.

### Study limitations

First, the relatively low number of events over 5 years of follow-up limits our power to assess multiple subgroups. This, in part, reflects our selection of the hard clinical endpoint of coronary heart disease death or nonfatal myocardial infarction as well as the low to intermediate risk of the population recruited in the SCOT-HEART trial. Second, the SCOT-HEART study was not designed or powered for this secondary analysis, and our findings are exploratory. Third, we used a simple and pragmatic approach to the identification of adverse plaques that would not greatly add to the time spent analyzing CT scans. Automated software that can identify plaque characteristics may further improve observer variability in the classification of adverse plaques and help standardization. Finally, the trial did encourage the use of secondary preventative therapies in patients found to have either obstructive or nonobstructive disease. This will have inevitably reduced the number of events as well as reduced our ability to identify associations between plaque characteristics and clinical events because they will have been modified by the initiation of antiplatelet and statin therapies.

## Conclusions

We have demonstrated that adverse plaque characteristics provide prognostic information out to 5 years, but that this is not independent of plaque burden assessed by coronary artery calcium score. Patients with obstructive disease and adverse plaques have the highest event rates throughout follow-up. This may aid the identification of a subgroup of patients who would benefit from more intensive medical therapy.

Perspectives **COMPETENCY IN MEDICAL KNOWLEDGE:** Specific characteristics of coronary artery atherosclerotic plaques are associated with a higher risk of death due to coronary heart disease or nonfatal myocardial infarction. This association is related to the severity of calcification, a marker of plaque burden.

**TRANSLATIONAL OUTLOOK:** Prospective studies should investigate whether more intensive therapy of stable patients with high-risk coronary plaque characteristics reduces the risk of clinical ischemic events.
