# Supplementary material for: Coronary Artery Plaque Characteristics Associated With Adverse Outcomes in the SCOT-HEART Study
Source: J Am Coll Cardiol. 2019 Jan 29;73(3):291–301. doi: 10.1016/j.jacc.2018.10.066 (PMC6342893; doi:10.1016/j.jacc.2018.10.066)

**Supplementary Material**

**Online Table 1: Frequency of adverse plaque in patients with non-obstructive and obstructive coronary artery disease in each of the 15 coronary artery segments.**

|  | **Patients with non-obstructive disease**  **N = 671** | | **Patients with obstructive disease**  **N = 452** | |
| --- | --- | --- | --- | --- |
|  | N | % | N | % |
| Left Main Stem | 19 | 2.8 | 41 | 9.1 |
| Prox LAD | 170 | 25.3 | 218 | 48.2 |
| Mid LAD | 81 | 12.1 | 153 | 33.8 |
| Dist LAD | 4 | 0.6 | 17 | 3.8 |
| D1 | 4 | 0.6 | 15 | 3.3 |
| D2 | 1 | 0.1 | 4 | 0.9 |
| Prox Cx | 28 | 4.2 | 81 | 17.9 |
| OM1 | 6 | 0.9 | 21 | 4.6 |
| AV Cx | 2 | 0.3 | 9 | 2.0 |
| OM 2 | 0 | 0 | 1 | 0.2 |
| Mid Dist Cx | 2 | 0.3 | 3 | 0.7 |
| Prox RCA | 34 | 5.1 | 82 | 18.1 |
| Mid RCA | 30 | 4.5 | 102 | 22.6 |
| Distal RCA | 9 | 1.3 | 42 | 9.3 |
| RCA/CX PD | 2 | 0.3 | 8 | 1.8 |

*Prox, proximal; RCA, right coronary artery; Cx, circumflex; PD, posterior descending; LM, left main; LAD, left anterior descending; D1, first diagonal; D2, second diagonal; OM1, obtuse marginal; AV, atrioventricular.*

**Online Table 2: Presence of positive remodelling and low attenuation plaque in different coronary artery segments.**

|  | **Positive remodelling AND low attenuation plaque** | |
| --- | --- | --- |
|  | N | % |
| Left Main Stem | 11 | 0.6 |
| Prox LAD | 75 | 4.2 |
| Mid LAD | 40 | 2.3 |
| Dist LAD | 3 | 0.2 |
| D1 | 1 | 0.1 |
| D2 | 0 | 0.0 |
| Prox Cx | 15 | 0.8 |
| OM1 | 3 | 0.2 |
| AV Cx | 1 | 0.1 |
| OM 2 | 0 | 0 |
| Mid Dist Cx | 0 | 0 |
| Prox RCA | 16 | 0.9 |
| Mid RCA | 17 | 1.0 |
| Distal RCA | 4 | 0.2 |
| RCA/CX PD | 1 | 0.1 |

*Prox, proximal; RCA, right coronary artery; Cx, circumflex; PD, posterior descending; LM, left main; LAD, left anterior descending; D1, first diagonal; D2, second diagonal; OM1, obtuse marginal; AV, atrioventricular.*

**Online** **Table 3: Frequency of fatal and non-fatal myocardial infarction at 30 days and 1 year in patients with and without adverse plaques**.

|  | **No adverse plaques**  **N = 1161** | **One or more adverse plaques**  **N = 608** | **P** |
| --- | --- | --- | --- |
| 30 days | 1  (0.1%) | 2  (0.3%) | 1.000 |
| 1 year | 6  (0.5%) | 9  (1.5%) | 0.053 |

**Online Table 4: Frequency of adverse plaques in patients with different numbers of coronary arteries with obstructive disease.**

| **Number of vessels with obstructive disease** | **Frequency of adverse plaque** |
| --- | --- |
| 1 vessel disease | 149 (72%) |
| 2 vessel disease | 99 (77%) |
| 3 vessel disease | 92 (79%) |

**Online Table 5: Characteristics of study participants with non-obstructive disease and obstructive disease with and without adverse plaque.**

|  | | **Non-obstructive disease** | | | **Obstructive disease** | | |
| --- | --- | --- | --- | --- | --- | --- | --- |
|  |  | **No adverse plaque**  **n=403** | **With adverse plaque**  **n=268** | **P-value** | **No adverse plaque**  **n=112** | **With adverse plaque**  **n=340** | **P-value** |
| Male | | 208 (52%) | 179 (67%) | **<0.001** | 74 (66%) | 273 (80%) | **0.002** |
| Age | | 59.0 ± 8.7 | 59.7 ± 8.2 | 0.282 | 61.4 ± 8.7 | 61.6 ± 7.4 | 0.823 |
| Body mass index (kg/m^2^) | | 30.0 ± 5.2 | 28.4 ± 4.9 | **<0.001** | 29.9 ± 5.3 | 29.2 ± 4.6 | 0.210 |
| Atrial fibrillation | | 7 (1.7%) | 5 (1.9%) | 1.000 | 1 (0.9%) | 9 (2.6%) | 0.463 |
| Smoking status | Current smoker | 66 (16%) | 62 (23%) | **0.019** | 26 (23%) | 60 (18%) | 0.393 |
|  | Ex-smoker | 145 (36%) | 105 (39%) |  | 38 (34%) | 131 (39%) |  |
|  | Non-smoker | 192 (48%) | 101 (38%) |  | 48 (43%) | 149 (44%) |  |
| Hypertension | | 157 (39%) | 86 (32%) | 0.070 | 53 (48%) | 149 (45%) | 0.565 |
| Diabetes | | 56 (14%) | 31 (12%) | 0.379 | 20 (18%) | 37 (11%) | 0.054 |
| Family history | | 177 (44%) | 111 (41%) | 0.229 | 43 (38%) | 147 (43%) | 0.553 |
| Previous CHD | | 32 (7.9%) | 32 (12%) | 0.084 | 24 (21%) | 71 (21%) | 0.902 |
| Anginal symptoms | Typical angina | 122 (30%) | 88 (33%) | 0.439 | 70 (63%) | 219 (65%) | 0.643 |
|  | Atypical angina | 103 (26%) | 75 (28%) |  | 19 (17%) | 64 (19%) |  |
|  | Non-anginal | 178 (44%) | 105 (39%) |  | 23 (21%) | 57 (17%) |  |
| ASSIGN | | 19.3 ± 11.4 | 19.4 ± 10.0 | 0.921 | 24.0± 11.7 | 23.0 ± 10.8 | 0.428 |
| Coronary artery calcium score (Agatston units) | | 33 [3, 108] | 129 [35, 307] | **<0.001** | 206 [42, 590] | 546 [196, 1188] | **<0.001** |

*N (%), mean±standard deviation or median [interquartile range]*

**Online Table 6: Univariable analysis for fatal or non-fatal myocardial infarction in patients with adverse plaque features on CCTA stratified by age, sex and body mass index.**

|  | | |
| --- | --- | --- |
|  | **Age < 60 years** | **Age ≥ 60 years** |
| **Adverse plaque features on CCTA** | 3.65 (1.54, 8.65)  p=0.003 | 2.44 (0.97, 6.13)  p=0.057 |
|  | | |
|  | **Male** | **Female** |
| **Adverse plaque features on CCTA** | 2.44 (1.14, 5.22)  p=0.021 | 3.27 (1.00, 10.71)  p=0.051 |
|  | | |
|  | **BMI < 30 kg/m^2^** | **BMI ≥ 30 kg/m^2^** |
| **Adverse plaque features on CCTA** | 1.76 (0.83, 3.74)  p=0.143 | 9.06 (2.53, 32.50)  p=0.001 |
|  | | |
|  | **Low cardiovascular risk score*** | **High cardiovascular risk score*** |
| **Adverse plaque features on CCTA** | 3.12 (1.35, 7.21)  p=0.008 | 2.64 (1.00, 6.94)  p=0.050 |

* High cardiovascular risk score defined as an ASSIGN score greater than or equal to 20.

*CCTA – Coronary Computed Tomography angiography; BMI – Body-mass index*

**Online Table 7: Per patient assessment of plaque characteristics and subsequent fatal or non-fatal myocardial infarction across the total population.**

|  | | **Patients**  **n (%)** | **Fatal or non-fatal myocardial infarction** | |
| --- | --- | --- | --- | --- |
|  |  |  | **n (%)** | **Hazard ratio**  **(**95% confidence intervals) |
| **Positive remodeling** | **Present** | 602 (34%) | 25 (4.1%) | 3.05 (1.63, 5.71)  P<0.001 |
|  | **Absent** | 1166 (66%) | 16 (1.4%) |  |
| **Low attenuation plaque** | **Present** | 168 (10%) | 6 (3.6%) | 1.60 (0.67, 3.80)  p=0.289 |
|  | **Absent** | 1601 (91%) | 35 (2.2%) |  |
| **Spotty calcification** | **Present** | 299 (17%) | 6 (2.0%) | 0.83 (0.35, 1.97)  p=0.669 |
|  | **Absent** | 1470 (83%) | 35 (2.4%) |  |
| **Napkin ring** | **Present** | 76 (4.3%) | 2 (2.6%) | 1.16 (0.28. 4.79)  p=0.841 |
|  | **Absent** | 1693 (96%) | 39 (2.3%) |  |

**Online** **Table 8: Cardiovascular risk and preventative medication use in patients with different severity of coronary artery calcification.**

|  | Coronary artery calcification (Agatston score) | | | | |
| --- | --- | --- | --- | --- | --- |
|  | 0 | 1-99 | 100-399 | >400 | P |
| Cardiovascular risk score * | 12.7 ± 8.7 | 18.6 ± 10.6 | 22.3 ± 11.0 | 24.1 ± 11.1 | <0.001 |
| Preventative medication use at 6 weeks | 315  46% | 337  73% | 271  89% | 292  93% | <0.001 |
| Adverse plaque | 16  2% | 148  32% | 203  67% | 241  77% | <0.001 |

* *ASSIGN score*

**Online Figure 1: CONSORT diagram.**
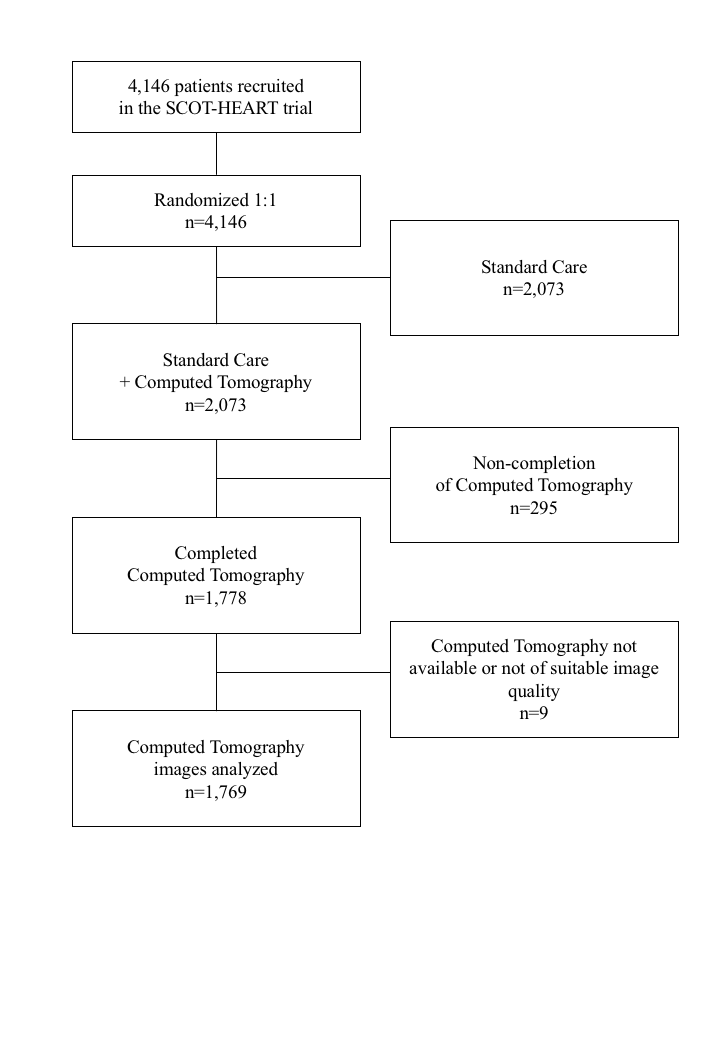

Supplement: Online Tables 1–8 and Online Figure 1 [file mmc1.docx]
